# Applications of Bioinorganic Nano‐Based Delivery Systems—Diagnostics and Therapeutics

**DOI:** 10.1002/ddr.70276

**Published:** 2026-08-03

**Authors:** Frank Ssengooba, Jubilee Andrew, Donald Fernandes

**Affiliations:** ^1^ Department of Pharmacy Practice, School of Pharmacy Sefako Makgatho Health Sciences University Pretoria South Africa; ^2^ Department of Pharmaceutical Sciences, School of Pharmacy Sefako Makgatho Health Sciences University Pretoria South Africa; ^3^ Department of Chemistry and Biology Toronto Metropolitan University Toronto Ontario Canada

**Keywords:** diagnostics, inorganic nanoparticles, nanomedicine, therapeutics

## Abstract

Nanotechnology has emerged as a promising avenue for producing nanomedicines as alternatives to conventional drugs. Many organic nanoparticles, such as liposomes used in formulations like Doxil, Onivyde, and Marqibo, are approved by the Food and Drug Administration (FDA) and instrumental in treating various types of cancers. However, due to the many challenges associated with these formulations, alternative delivery systems have been explored, particularly those derived from inorganic sources. In particular, bioinorganic nano‐based delivery systems (BNDSs) such as gold, silver, platinum, silicon‐based, metal oxide and hybrid nanoparticles have been found to be useful in treating cancer and infectious and noncommunicable diseases. This review presents the most commonly used BNDSs, providing an in‐depth discussion on their use as therapeutic and imaging agents, the challenges associated with their use and current trends and future perspectives in their development for enhancing efficacy.

## 1 Introduction

The recent surge in publications on nanoparticles indicates that nanotechnology is revolutionizing various aspects of everyday life. In the last few decades there has been cutting‐edge nanotechnology research in the medical, energy, food and environmental science fields, showing immense potential for addressing current challenges. For example, nanoparticles can be used for environmental remediation, energy storage and medicine, due to their enhanced reactivity, high surface area‐to‐volume ratio and tuneable optical and electrical properties (Fernandes 2022a, 2023b, 2023c; Rahman et al. 2023). Advancements in nanotechnology have led to overcoming challenges associated with delivery of free drug formulations. Research on nanoparticles as safe, efficient and reliable targeted drug delivery systems is extensive. The development of such nanoparticles enables the ability to improve the treatment and diagnosis of various diseases by minimizing side effects. For instance, the recent COVID‐19 pandemic provided an opportunity to explore lipid nanoparticles for vaccine development and delivery (Chauhan et al. 2020; Tang et al. 2021; Yang 2021). Organic nanomaterials have mainly received attention in drug delivery, with liposomal drug formulations being the first nanoparticle‐based therapeutic agents to be approved by the FDA.

Despite the successful applications of liposomes in drug delivery, several challenges and concerns exist, relating to scalability in production, immunogenicity and their drug release profiles (Gyanani et al. 2021). BNDSs, which utilize nanoparticles synthesized from inorganic materials such as gold, silver, iron and zinc, to deliver therapeutic molecules, present a promising alternative. Inorganic nanoparticles can provide better drug loading capacity compared to organic nanoparticles and exhibit excellent stability and tuneable degradation rates (Unnikrishnan et al. 2023). Metal ions have important functions in gene transcription, oxidative stress and enzymatic action. Structurally, bioinorganic nanoparticles comprise an inorganic metal‐based core stabilized by biological molecules such as lipids, proteins and polysaccharides (Naseem et al. 2024; Paul and Sharma 2020). Heavy metals, including iron (Fe), gold (Au), silver (Ag), platinum (Pt), and metal oxides like iron oxide and titanium dioxide, to name a few, have been utilized in fabrication of nanoparticles for drug delivery. Therapeutic agents can be loaded within the core or attached through surface modification for inorganic nanoparticles. Nanoparticle versatility, in terms on their size, shape, and optical properties, is crucial for theranostic applications, for therapy and imaging (Khan et al. 2019). Unlike conventional drugs, bioinorganic nanoparticles can be used for site‐specific targeting and controlled drug delivery, with significantly reduced systemic toxicity and improved patient outcomes. They interact with proteins and lipids in the body (e.g., cellular components), eliciting physiological changes for disease diagnosis and treatment (Augustine et al. 2020; R. Huang et al. 2021). Several factors of inorganic nanoparticles, including their size, shape, protein corona, and surface modification are important when interacting with biological systems.

The focus of this review is on exploring the main inorganic nanoparticles for bio‐imaging and therapy, and their important properties and applications for a wide range of diseases. Results from in vitro, in vivo and clinical studies are discussed to demonstrate their effectiveness for theranostics. It serves as an important comprehensive source for those studying in relevant research areas and those working in translational research interested in nanotechnology and inorganic nanoparticles.

## 2 Properties of Bioinorganic Nanomaterials for Effective Drug Delivery

The emergence of precision medicine has contributed to the development of new drug delivery techniques, for overcoming challenges such as adverse drug effects and resistance faced by conventional treatments (e.g., chemotherapy, radiation, surgery). Precision medicine aims to optimize drug therapy by considering an individual's genetic profile and drug metabolism pathways rather than significant nonspecific targeting of both disease and healthy sites. In this regard, nanomaterials such as metallic nanoparticles offer the potential for applications in precision medicine. The unique properties of nanomaterials synthesized using inorganic elements, important for biomedical applications, are discussed in detail below, with their advantages, limitations, applications and clinical status presented in Table 1.

**Table 1:** Comparison of inorganic nano‐based systems used for biomedical applications.

Type of nanoparticles	Advantages	Limitations	Applications	Clinical status	References
Noble metal nanoparticles	Generally biocompatible Homogeneity in size, shape, and surface properties Intrinsic properties (e.g., absorption, scattering) for enhancing diagnostic imaging and therapy	Lower biodegradability Tendency to aggregate and be unstable in certain aqueous environments Potential toxicity in healthy cells via deoxyribonucleic acid (DNA) damage and increased lactate dehydrogenase leakage Can inhibit proliferation of stem cells	Optical imaging Photothermal therapy Radiation therapy Antioxidant Antimicrobial Wound healing	Clinical trials involving nanogold‐based platforms such as AuroLase (NCT02680535, NCT04240639, NCT00848042), NU‐0129 (NCT03020017) and CNM‐Au8 (NCT04098406, NCT05299658, NCT04414345), for ablation therapy, gene therapy and treatment of neurodegenerative diseases, respectively Clinical trials using formulations of silver nanoparticles (AgNPs) for enzymatic therapy (NCT01243320, NCT01405794) and treatment of microbial pathogens (NCT02403479, NCT02761525, NCT03752424), virus‐caused plantar warts (NCT02338336), COVID‐19 infections (NCT04894409, NCT04978025), wounds (NCT04834245), and skin conditions (NCT05666011, NCT03039634) Research phase for platinum nanoparticles (PtNPs) even though platinum‐based drugs, including cisplatin, carboplatin and oxaliplatin, have been used in clinical trials and are FDA approved for treating various cancers	Almanza‐Reyes et al. (2021); Biosciences (2016); Jue et al. (2022); Kadria‐Vili et al. (2024); Kumthekar et al. (2021); Munger et al. (2014); Schram et al. (2017); J. R. Scott et al. (2017); Vucic et al. (2021); Vucic et al. (2023); Yahia et al. (2021); Yousefpour et al. (2023)
Metal oxide nanoparticles	Simple preparation methods Can be functionalized easily Possible to incorporate into hydrophilic and hydrophobic systems Controlled delivery for bio‐sensing and treatment due to magnetic properties	Alteration of conductance due to adsorption reactions and temperature changes, affecting biomedical applications Scalability issues related to cost, yield and control over particle size and shape	Magnetic resonance imaging (MRI) Magnetic hyperthermia Radiation dose enhancement Antidiabetic Anti‐inflammatory Antioxidant Antibacterial Photocatalytic Iron deficiency anemia	Hafnium oxide (Hensify) approved by the European Medicines Agency (EMA) as a radioenhancer for treatment of locally advanced soft tissue sarcoma Several formulations made of superparamagnetic iron oxide nanoparticles such as Feridex IV, Resovist, Combidex, Sinerem, and VSOP‐C184, have been clinically tested as magnetic resonance contrast agents for the liver, lymph nodes, and blood vessels. Feraheme is FDA approved for the treatment of iron deficiency in adult chronic kidney disease patients. NanoTherm approved by FDA and EMA for magnetic hyperthermia using heat generated from iron oxide nanoparticles to destroy tumor cells Other types of metal oxide nanoparticles such as zinc oxide, titanium dioxide, copper oxide, magnesium oxide, and cerium oxide nanoparticles have shown antidiabetic, antimicrobial, anti‐inflammatory, and anticancer activities in preclinical studies. These nanoparticles have not got much attention for clinical studies as detailed biocompatibility and toxicity studies have not been carried out.	Bonvalot et al. (2017); Hetzel et al. (2014); M. Johannsen, Gneveckow, Taymoorian, et al. (2007); Reimer and Balzer (2003); Ros et al. (1995); Team (2006); Wu et al. (2011)
Silicon‐based nanoparticles	Ease of synthesis Can modify surface for carrying drugs and/or imaging agents Inert chemical composition	In vivo immunotoxicity Difficulty in controlling size Some synthesis methods can lead to generation of unwanted by‐products	Nuclear imaging Photothermal therapy Controlled drug release	Amorphous silica is generally recognized as safe by several regulatory authorities such as Therapeutic Goods Administration, EMA, and FDA. Silica‐based nanoparticles have entered clinical trials for a variety of biomedical applications, including oral drug delivery, diagnostics, and photothermal ablation therapy. Nanoparticles such as silica core‐shell nanoparticles (Cornell dots) have been used as fluorescent and positron emission tomography tracers for tumors, such as melanoma or malignant brain tumors (NCT03465618, NCT01266096, NCT02106598). These nanoparticles can be labeled with ligands for active targeting. Mesoporous silica nanoparticles can be used to enhance the bioavailability of poorly soluble drugs such as fenofibrate. The clinical trial was conducted by SGS Life Science Services.	Janjua et al. (2021); Phillips et al. (2014); Zanoni et al. (2021)

### 2.1 Intrinsic Activity

Recent innovative techniques have provided treatment dosage forms that are superior to conventional dosage forms, with noble metals possessing biological activity (Dutta et al. 2024). Metals such as gold, silver and platinum have been long used for their antimicrobial and anti‐neoplastic properties (Mathur et al. 2018; Yamada et al. 2015). Their biological activity forms the basis on which newer dosage forms are developed. Metal nanoparticles are designed to enhance the biological activity of metals by improving bioavailability and imparting favorable physicochemical properties for therapy, while reducing systemic toxicity (Slavin et al. 2017). Formulations of silver nanoparticles are used as broad‐spectrum biocides, whose free Ag^+^ increase anti‐microbial potency via production of free radicals and suppression of DNA replication (Rodrigues et al. 2024). Biosynthesized AgNPs can be combined with antibiotics for synergistic activity against various microorganisms, for managing infectious diseases (Haji et al. 2022). Gold, silver and platinum nanoparticles exhibit the ability to overcome drug resistance, unlike many chemotherapy drugs. These noble metal‐based nanoparticles can spontaneously absorb glutathione (GSH), with the sulfhydryl group as a capping agent. This feature is important for cancer therapy since cancer cells need GSH to protect themselves from oxidative stress and to support their rapid growth and proliferation (Zeng et al. 2020).

### 2.2 Enhanced Cellular Uptake

**Figure 1:**
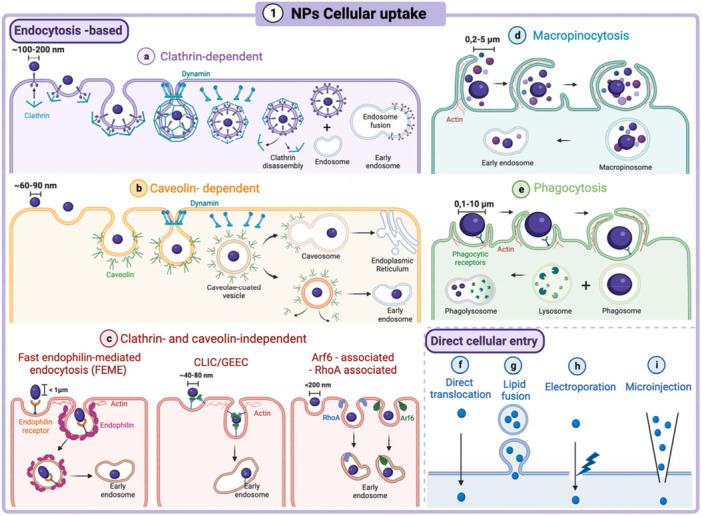
Schematic representation showing the mechanisms of nanoparticle cellular internalization. Nanoparticles can be internalized through the endocytosis–based mechanism (left), which includes (a) clathrin‐dependent; (b) caveolin‐dependent; (c) clathrin‐ and caveolin‐independent; (d) macropinocytosis; and (e) phagocytosis; and through direct cellular entry (right), which includes (f) direct translocation, (g) lipid fusion, (h) electroporation, and (i) microinjection. Reproduced from Toscano and Torres‐Arias 2023, Copyright (2023), Elsevier (https://creativecommons.org/licenses/by‐nc‐nd/4.0/).

The field of pathophysiology has provided evidence that diseases often arise from cellular‐level disruptions, impacting molecular and genetic processes (Shull et al. 1992). To treat and diagnose diseases (e.g., cancer), infections and genetic disorders, drug delivery systems are able to transport the payload into cells. Genetic diseases such as cancer are influenced by changes in genetic material situated inside cells. To correct genetic defects, nanomaterials must traverse cellular membranes. Cellular uptake is crucial for ensuring therapeutic activity of nanoparticles. It is a complex process influenced by factors such as the physicochemical properties of nanoparticles and the cellular environment. The enhanced cellular uptake of BNDSs is attributed to properties such as size, shape, surface charge and biocompatibility, and the ability of bio‐functionalization for nanoparticles. Endocytosis is the primary cellular uptake mechanism of BNDSs. Generally, endocytosis involves the engulfing of nanoparticles, using vesicles to transport them into the cell (Figure 1). The cellular uptake mechanisms for nanoparticles are further complicated by the physical state, whether solid or fluid, and the size of nanoparticles. Phagocytosis and pinocytosis are forms of endocytosis which involve cell engulfing and internalization of particles (Foroozandeh and Aziz 2018).

#### 2.2.1 Size

The size of nanoparticles for dosage forms is important for ensuring high efficacy. The small size of bioinorganic nanoparticles enables them to have a high surface area‐to‐volume ratio, for loading significant amounts of therapeutic agents for drug delivery. Size affects the biological activity and biosafety of bioinorganic nanoparticles. It is a critical factor for the successful delivery of bioinorganic nanomaterials into cells, impacting cellular uptake and trafficking (Foroozandeh and Aziz 2018). Bioinorganic nanoparticles must traverse the cell membrane and be delivered intracellularly via specialized receptors. Studies have shown that bioinorganic nanomaterials in the size range of 40–50 nm have the highest intracellular uptake compared to those with sizes falling outside this range. A study conducted with gold nanoparticles showed the inability of small particles to bind to membrane receptors for cellular uptake while larger particles could not be sufficiently wrapped by the cell membrane for uptake (Behzadi et al. 2017). Ultrasmall nanoparticles less than 10 nm in size easily pass through the glomerular filtration barrier in the kidneys, leading to rapid urinary excretion and low accumulation in the liver and spleen. Larger particles (i.e., greater than 100–200 nm) are quickly recognized by opsonin proteins in the blood, leading to rapid sequestration by the reticuloendothelial system, specifically in the liver (i.e., Kupffer cells) and spleen. Micrometer particles (i.e., > 1 µm) are typically filtered by the lungs or trapped in the spleen, remaining in the body for long periods.

While it's well‐established that the size of nanoparticles impacts their biological performance, recent studies have revealed interesting findings. For example, Bélteky et al. explored the effects of particle size of AgNPs at biological conditions. Their study investigated AgNPs of varying diameters (i.e., 10, 20, and 50 nm). They found that larger particles showed increased resistance to aggregation while retaining biological activity. The results indicate that aiming for the smallest possible nanoparticles might not be the best course of action (Bélteky et al. 2021).

#### 2.2.2 Shape

Bioinorganic nanoparticles of various shapes, including spheres, cubes, tubes and rods, have been reported for drug delivery, with different efficiencies (Augustine et al. 2020; Slavin et al. 2017). Spherical nanoparticles possess excellent colloidal stability and can be easily functionalized, having many more applications reported, compared to other nanoparticles with different shape. Recently, the applications of nanorods and nanotubes have been explored owing to their stability, excellent conductivity and high surface area‐to‐volume ratio. Cellular uptake of metallic nanoparticles based on shape does not conform to a one‐shape‐fits‐all relationship. For example, certain studies have demonstrated very efficient uptake of spherical gold nanoparticles (AuNPs). However, in some cases other shaped nanoparticles such as gold nanostars can exhibit highest cellular uptake, even compared to other structures such as gold nanorods and nanocages (Pakravan et al. 2021). The work of Rotz et al. revealed efficient uptake of gold nanostars for delivering gadolinium (III)‐DNA contrast agents for MRI, with superior cellular uptake over spherical nanoparticles (NPs) (Rotz et al. 2015). The difference in Gd(III) delivery is primarily attributed to greater loading as a result of the higher surface area of the gold nanostars compared to the nanospheres. The shape of nanoparticles profoundly affects cellular internalization by influencing how they interact with cell membranes and the various endocytic pathways involved (Hadji and Bouchemal 2022). Spherical nanoparticles are often efficiently internalized via clathrin‐mediated endocytosis, while nonspherical nanoparticles can use alternative mechanisms such as macropinocytosis or phagocytosis. Elongated or high aspect‐ratio (AR) nanoparticles can exhibit increased margination and adhesion to cell surfaces, promoting cell capture, but can also show reduced penetration. Ultimately, shape influences the contact area with the cell, the energy required for internalization, and the specific internal pathway, which varies depending on the dimensions of the nanoparticles and the cell's machinery (e.g., cytoskeletal proteins). Nonspherical nanoparticles, including those that are inorganic, with high ARs, also have favorable pharmacokinetics, with prolonged circulation times in the blood and slower elimination rates, compared to spherical nanoparticles, due to lower uptake by macrophages and weaker interactions with proteins involved in opsonization (Chu et al. 2013; Arnida et al. 2011; Muro et al. 2008).

#### 2.2.3 Surface Charge

Surface charge plays a crucial role in nanomaterial uptake into cells, where bioinorganic materials are delivered for cellular imaging, drug and/or gene delivery (Foroozandeh and Aziz 2018). Research has shown superior cellular uptake of charged particles over uncharged particles. Additionally, a better uptake was observed for positively charged particles over negatively charged ones, illustrating the crucial role of surface charge (Marano et al. 2011; Panariti et al. 2012). Cell membranes are negatively charged giving higher possibilities of anchoring of positively charged nanoparticles to the cell surface for endocytosis. Surface charge is widely regarded as a main determinant of interaction between NPs and cells, even in the presence of a protein corona (Barbalinardo et al. 2025). While the protein corona forms rapidly in physiological conditions and surrounds the original surface of the nanomaterial, studies show that surface charge often dictates the composition of this corona and continues to govern cellular uptake and biodistribution.

## 3 General Use of BNDSs for Diagnosis and Therapy

Inorganic NPs possess physicochemical properties that enable them to interact with biological systems. BNDSs incorporate inorganic materials to facilitate targeted theranostics, by enhancing effectiveness of molecules for therapy and imaging. BNDSs offer a promising approach to enhance drug delivery by leveraging the unique properties of inorganic materials by improving solubility, bioavailability and stability of drugs (Alshammari et al. 2023). Nanoparticles made using gold (Au), silver (Ag), silicon (Si) and iron oxide have shown promise in biomedical applications (Akif S et al. 2022; Sánchez‐López et al. 2020). Many chemotherapeutic drugs exhibit low aqueous solubility. These drugs can be incorporated with inorganic NPs for enhancing their absorption in biological fluids (e.g., blood) by reducing their biodegradation rate (H. Li et al. 2023). Magnetic and optical properties of inorganic NPs make it easier to track their movement within the body and enable a better understanding of their pharmacokinetics and bio‐distribution. Numerous BNDSs are designed to integrate nanomedicines with imaging agents (e.g., fluorescence, MRI contrast agents), allowing for real‐time monitoring of drug distribution and effectiveness in vivo.

BNDSs are designed with biocompatible and biodegradable materials for safe clearance from the body, important for reducing toxicity and side effects of many drugs. To improve therapeutic effects, BNDSs can be utilized for delivering multiple compounds in one platform such as hydrophobic drugs, proteins, DNA and ribonucleic acid (RNA) materials (Walia and Mehta 2024). They exhibit sustained drug release, reducing dosing frequencies and the need for higher doses (Chen et al. 2016).

Several properties of metal, metal sulphides and metal oxides such as shape, porosity, particle size, stability, surface modification and charge dispersion, have an impact on their therapeutic efficacy. The ability to control various parameters of these materials make them attractive for therapeutics. Magnetic, thermal, optical and electrochemical characteristics along with functionalization of NPs are used to deliver genetic material and drugs precisely at the targeted site(s) (Jin et al. 2024; Pandey and Dahiya 2016; Walia and Mehta 2024). BNDSs can be functionalized with ligands that specifically target certain cells, minimizing off‐target delivery and unwanted side effects. They can also be designed to release their payloads in response to internal and/or external stimuli such as temperature changes, pH variations, laser irradiation and the presence of certain biomolecules (Adepu and Ramakrishna 2021). Furthermore, synergistic effects can be achieved through delivery of multiple chemotherapeutic and/or immunotherapeutic agents using a single carrier. This approach can enhance therapeutic outcomes (Johnson et al. 2020).

Bioinorganic nanoparticles have been studied to investigate their ability to be applied in DNA vaccine delivery systems, for improving immunogenicity, compared to conventional methods. Bioinorganic NPs can serve as adjuvants in vaccine delivery to enhance immune response and enable controlled release. They can be engineered to deliver antigens or genetic material that stimulates an immune response against specific tumor antigens, enhancing the efficacy of tumor vaccines (Johnson et al. 2020; Yi et al. 2023). AuNPs play a significant role in nanovaccine development as they possess excellent immune‐modulatory and adjuvant characteristics. AuNPs are relatively inert, easy to modify/functionalize and have low toxicity, rendering them suitable candidates for the delivery of drugs or antigens. AuNPs have been modified to detect and diagnose a wide range of infections. Ongoing studies are being carried out to investigate the use of BNDSs in gene therapy applications. For example, cationic lipid‐coated AuNPs have proven to be promising agents for the delivery of nucleic acids. The loading of genetic material for these NPs improves their stability and is used for their controlled release (Graczyk et al. 2021; Sengupta et al. 2022).

## 4 Common BNDSs Used for Diagnostic and Therapeutic Applications

**Figure 2:**
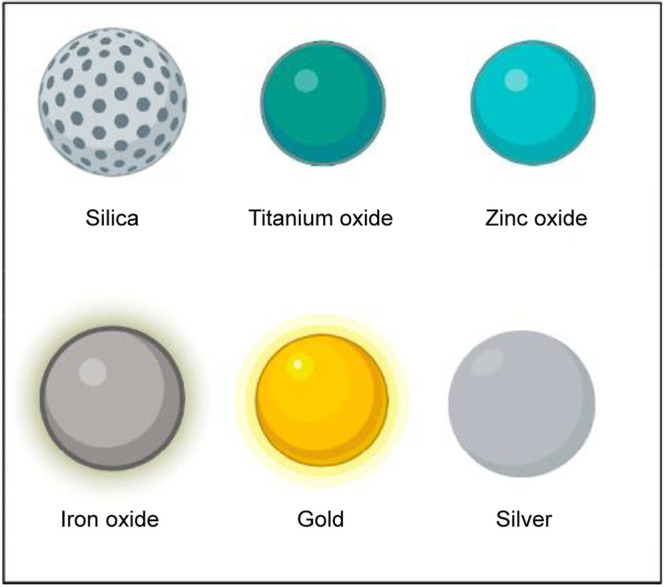
BNDSs for theranostics. Important inorganic nanoparticles that can be used for therapy and bio‐imaging include silica, titanium oxide, zinc oxide, iron oxide, gold and silver NPs due to their superior structural and mechanical properties and unique electrical, magnetic and optical properties. Reproduced from Eker et al. 2024, Copyright (2024), MDPI (https://creativecommons.org/licenses/by/4.0/).

Many BNDSs for diagnostic and therapeutic applications include metal‐based nanoparticles (Ozdal and Gurkok 2022; B. Wang et al. 2024). Metal ions are used for construction and can be directly used for different therapies such as phototherapy and chemotherapy. Nanoparticles containing more than one type of metal, can be used as contrast agents for cancer imaging. Conjugation can significantly improve drug loading, stability and biocompatibility of BNDSs. Some of the commonly used inorganic nanoparticles are shown in Figure 2 and discussed in the following subsections.

### 4.1 Gold Nanoparticles

Challenges encountered with conventional treatments, such as drug and antibiotic resistance, has led to exploring AuNPs as delivery systems. AuNPs stand out among the different types of nanoparticles, as one of the most developed for cancer drug delivery. The facile synthesis, versatile sizes and shapes, bio‐functionalization capability, excellent surface properties and photothermal properties make them ideal for cancer theranostics (Fernandes 2024a; Yafout et al. 2021). Extensive studies have been carried out on the use of gold to treat several diseases in various regions of the world, leading to investigations on the use of colloidal gold. For example, Selim and Hendi showed that AuNPs can induce apoptosis in cancer cells and exert concentration‐dependent cytotoxicity via p53, bax/bcl‐2 and caspase pathways (Selim and Hendi 2012).

Metallic nanoparticles synthesized using gold, silver, and copper exhibit a unique phenomenon termed localized surface plasmon resonance (LSPR) that has been used to design biosensors for diagnostic purposes. Upon light illumination, the free electrons on the metallic nanoparticle surface collectively oscillate, resulting in a potent electromagnetic field and improved optical properties (Mcoyi et al. 2024; Sui et al. 2019). Plasmonic enhancements in biological environments are heavily modulated by the geometries (e.g., shape, size, assembly) of nanostructures and the surrounding dielectric environment (e.g., refractive index of media). Larger and/or more polydisperse particles often exhibit red‐shifted and broadened LSPR peaks. Increased refractive index of the surrounding medium causes a red‐shift of the LSPR peak, while particles with sharper edges/corners lead to increase in local electromagnetic fields, strengthening sensitivity (Guo et al. 2015). These properties are explored to study interactions between ligands and receptors and DNA and RNA. For their biosensing abilities in disease diagnosis, the optical properties of AuNPs have been utilized to detect infectious diseases (Guliy and Dykman 2024). For example, Waitkus et al. designed a novel LSPR system based on the coupling of gold nanomushrooms and AuNPs, important in molecular diagnostics, with the ability for CRISPR‐Cas13a based RNA sensing (Waitkus et al. 2023).

### 4.2 Silver Nanoparticles

Silver is another noble metal, used to design novel delivery systems for theranostics, having antimicrobial and anticancer properties. Numerous studies have been reported employing safer green synthesis methods for AgNPs, with outstanding attributes. The excellent optical properties, high surface area‐to‐volume ratio and broad pharmacological activities make AgNPs attractive for use in various dosage forms. Jadhav et al. reported the successful synthesis of gels containing AgNPs, having remarkable anti‐bacterial activity against bacteria responsible for infections in burns (Jadhav et al. 2016). Silver nanoparticles, like gold nanoparticles, exhibit excellent biosensing properties due to the LSPR, albeit with superior sensitivity (Loiseau et al. 2019). This ability of AgNPs to interact with light has been used for theranostic purposes in the medical field. Zhao et al. designed a reusable biosensor utilizing AgNPs capable of detecting squamous cell carcinoma antigens for diagnosis (Zhao et al. 2014).

The improved efficacy of many drugs has been one of the main reasons for the frequent use of formulations containing nanoparticles. Haji et al. explored the synergistic anti‐bacterial activity of biosynthesized AgNPs against carbapenemase‐producing Gram‐negative bacilli (Haji et al. 2022). AgNPs with a size of 10 nm were successfully produced by *Acinetobacter baumannii* with minimum inhibitory concentrations (MICs) ranging from 8 to 64 μg/mL. Most significant, however, was the activity when AgNPs were used in combination with an antibiotic (*i.e*., imipenem, ceftriaxone, cefepime or ceftazidime) with fractional inhibitory concentrations between 0.13 and 0.56, requiring significantly lower amounts of antibiotic for achieving the same response (*e.g*., from ≥ 1024 μg/mL for ceftriaxone alone to 4 μg/mL for AgNPs + ceftriaxone). This study reveals the promising ability of combined therapy involving nanoparticles and antibiotics for managing infectious diseases.

The high prevalence of inflammatory diseases is a significant concern, with many anti‐inflammatory drugs having undesirable side effects, which can limit their use in patients. In this regard, research has focused on possible nanoparticle‐based formulations for better managing inflammatory disorders. Nanodrug delivery to treat inflammatory conditions of the gastrointestinal tract (GI) is promising and was investigated in a study by Todorova and co‐workers (Todorova et al. 2023). They reported the synthesis of AgNPs for delivering 3‐methyl‐1‐phenylbutan‐2‐amine, a mebeverine precursor (MP), used for treating inflammatory bowel disease. The MP‐loaded AgNPs produced had a size, zeta potential and drug release of 18 nm, −10.72 ± 0.74 mV and 85%, respectively. This study provided a glimpse into the use of AgNPs for drug delivery to the GI in order to manage inflammatory bowel disease.

### 4.3 Platinum Nanoparticles

PtNPs possess several excellent attributes that have led to their exploration in drug delivery applications. In addition to their anticancer properties, PtNPs exhibit deep tissue penetration with low scattering and high absorption at near‐infrared wavelengths, causing extreme heating for thermoplasmonic applications (*e.g*., in nanomedicine, biomedical engineering) (Samadi et al. 2018). The discovery of the anti‐cancer properties of platinum by Barnett Rosenberg and colleagues in the 1960s is one of the major discoveries in the field of cancer therapy (Q. Zhang et al. 2023). Ever since this discovery, platinum‐based drugs (*e.g*., cisplatin) are commonly used in chemotherapy for treating different advanced cancers. Their cytotoxicity is attributed to the ability to cross‐link DNA, inhibiting its synthesis and function and eventually leading to apoptosis (Sahoo et al. 2024; Xie et al. 2021). Although platinum drugs have been successful in treating various solid tumors, they are associated with systemic toxicity and cancer cell resistance. This has led to the exploration of platinum‐based nanosized carriers for overcoming these obstacles. A significant amount of literature is available on strategies to modify platinum‐based drugs and carriers to maximize their excellent anti‐neoplastic activity.

To overcome clinical challenges, PtNPs have been designed for delivery of a combination of drugs. PtNPs offer a mechanism through which a combination of drugs can be used for treating cancer and infectious diseases, allowing for reduced drug dosages and attenuating side effects and cell resistance (R. Zhang et al. 2017). Surface modification of PtNPs *via* conjugation with various moieties such as polymers, antibodies, peptides, small molecules and proteins, can enhance circulation time and controlled release. Moreover, nanoparticles with bimetallic combinations of noble metals such as Pt‐Au can also be constructed to enhance properties (*e.g*., optical, magnetic) for improving therapeutic efficacy (Mandal 2024). Stavropoulou et al. reported on the facile synthesis of a hemocompatible bimetallic nanosystem consisting of gold nanoparticles coated with platinum nanoparticles, delivering a quinazoline derivative (Au@Pt@Q NPs) for theranostic purposes in glioblastoma. Au@Pt@Q NPs exhibited superior cytotoxic activity against glioblastoma cancer cells (*i.e*., U87‐MG) when compared to Au‐only nanoparticles (Stavropoulou et al. n.d.).

Zhang et al. reported on the synthesis of nanoparticles co‐loaded with gemcitabine (GEM) and platinum (Pt) prodrugs *via* the layer‐by‐layer (LbL) method, with the potential for lung carcinoma treatment (R. Zhang et al. 2017). The LbL NPs were prepared by using chitosan‐Pt (CH‐Pt) as positively charged core and hyaluronic acid‐GEM (HA‐GEM) as negatively charged shell. The HA‐GEM/CH‐Pt NPs produced had a size of 187 nm, zeta potential of −21 mV and a drug loading efficiency of 90%, with sustained drug release. The HA‐GEM/CH‐Pt NPs showed superior *in vitro* and *in vivo* antitumor activity in NCl‐H460 cells compared to nanoparticles with only one drug loaded. The results reveal the synergistic anti‐neoplastic effects of the NPs loaded with the prodrugs, CH‐Pt and HA‐GEM, for applications in cancer treatment.

The ability of platinum‐based drugs such as cisplatin, carboplatin, oxaliplatin and nedaplatin to form PtNPs after intravenous injection is an intriguing finding. A study by Zeng et al. investigated this ability and found that platinum‐based drugs are converted into PtNPs, with their surfaces covered by a protein corona when in circulation (Zeng et al. 2020). The *in vivo* biosynthesis of PtNPs was elucidated to be a result of the nucleation and growth processes, characterized by initial formation of weak bonds between the drug and albumin and subsequent growth in blood. The PtNPs had excellent and improved anticancer activity. This study sets the groundwork for further investigations into self‐assembled PtNPs for effective anti‐cancer treatment.

### 4.4 Silicon‐Based Nanoparticles

Silicon‐based nanoparticles such as silica nanoparticles (SNPs), and especially those that are mesoporous (MSNs), are used for biomedical applications due to their biocompatibility, tuneable properties and ability to deliver biomolecules. Their ability to be easily modified with other materials and high loading efficiency enable them to be used in drug delivery, diagnostic imaging and biosensing.

SNPs are used to improve the permeability of poorly water soluble drugs and their bioavailability, for improving therapeutic outcomes. Elbialy et al. fabricated smart MSNPs for the natural and fluorescent hydrophobic anticancer drug curcumin (Cur) (Elbialy et al. 2020). After 6 h incubation, intracellular fluorescence from free‐Cur was weak and its accumulation was decreased for HepG2 liver cancer cells, while those treated with PEG‐MSNPs‐Cur showed strong fluorescence, reflecting the high accumulation of nanocarriers inside cancerous cells. PEG‐MSNPs‐Cur was rapidly taken up by HeLa cervical cancer cells in the first half an hour. At 36 μg/mL Cur concentration, PEG‐MSNPs‐Cur showed cell viability less than 10% for HepG2 cells and less than 30% for HeLa cells after 24 h, which then considerably decreased to about 7% for both cancer cell types after 48 h. For the cells treated with free‐Cur with the same concentration (*i.e*., 36 μg/mL), the minimum cell viability did not exceed 76% after 48 h incubation. The half‐maximal inhibitory concentration (*i.e*., IC_50_) for HepG2 and HeLa cells treated with PEG‐MSNPs‐Cur were 20 and 28 μg/mL, respectively. The higher therapeutic efficacy of PEG‐MSNPs‐Cur over that of free‐Cur is due to the rapid internalization and accumulation of the nanocarrier inside tumor cells and enhanced release of Cur at acidic pH environment inside cells.

Silicon‐based nanoparticles can be used to carry peptides for effectively treating lung infections. Kwon et al. loaded tandem peptide anti‐infectives into the pores of silicon nanoparticles (*i.e*., peptide‐pSiNP) to reduce *Pseudomonas aeruginosa* bacterial numbers (Kwon et al. 2017). At 2 × 10^5^ CFU per mouse, development of lung infection with *P. aeruginosa* was aggressive, with only 10%–20% 24 h survival without therapeutic intervention. The peptide‐pSiNP formulations, with two doses of peptide‐pSiNPs at 30 μg of pSiNPs and 1.5 nanomoles peptide given, greatly improved the survival to 24 h. A lower than 20% survival was observed with vehicle treatment, and this increased to 100% survival with the peptide‐pSiNP formulation. Treatment of mice with empty pSiNPs appeared to cause a decrease in number of bacteria in the lung, but the difference to the control treatment was not statistically significant. However, a dramatic decrease in bacterial count was observed when peptide‐pSiNPs were administered, with lung titers 4–6 log_10_ lower than when there was no therapeutic intervention. All strains evaluated were susceptible to the tandem peptide construct and displayed MIC values between two‐ and four‐fold larger than the MIC for the PA01 laboratory strain.

Functionalized SNPs can be used for overcoming gastrointestinal barriers, which is important for treating diseases such as diabetes. Gao et al. utilized deoxycholic acid (DC)‐modified MSN, coated with sulfobetaine‐12 (SB‐12), to deliver insulin (Gao et al. 2021). The DC modified carrier could increase the absorption of loaded insulin in all intestine segments. After administering insulin formulations into diabetic rats, MSN‐DC@SB12 significantly induced hypoglycaemic effect and reduced the blood glucose level to 45% after 1 h administration. After 6 h, MSN‐DC@SB12 reached similar blood glucose level when compared to subcutaneously administered insulin group. These results indicate that insulin could gradually be released from the DC coated carrier, diffusing through the mucosal barrier rapidly. In addition, silica nanoparticles can be functionalized to carry molecules for various imaging (*e.g*., ultrasound, MR, optical, positron emission tomography) and treatment (*e.g*., phototherapies, radiation, magnetic hyperthermia) modalities (Liberman et al. 2014).

MSNs are highly versatile and stable drug carriers with tremendous potential for treating several diseases of different severities, due to their pore architecture, surface chemistry and release kinetics. Pore diameters (*i.e*., usually 2–50 nm) can be adjusted to accommodate different molecules, from small‐molecule drugs to large proteins and nucleic acids, with large pore volumes allowing for high drug loading and entrapment efficiency. Ordered, rigid, and well‐defined pore structures ensure uniform drug distribution and predictable release, while unordered structures can be used for faster release of therapeutics. Peng et al. prepared several different types of MSNs (*e.g*., hexagonal pore MSN, worm‐like pore MSN, hollow MSN, MSN‐PDA, expanded pore MSN) loaded with the model drug doxorubicin (DOX), and investigated DOX loading and their release performance, investigating the influence of structure and functional groups on the release performance of MSNs (Peng et al. 2022). DOX load was positively correlated with the specific surface area, with surface modification leading to a lower DOX load. After 36 h of DOX release, the hollow MSNs exhibited the highest cumulative DOX release (*i.e*., 78.52%) in acidic environments (*i.e*., pH 5), closely resembling disease sites, while the worm‐like pore MSNs exhibited the lowest cumulative DOX release (*i.e*., 35.90%). However, in terms of pH response, burst release rate and sustained release effect, hexagonal pore MSN‐NH_2_ and MSN‐PDA provided better overall release performance. The results showed that the selection of MSNs with suitable pore types and reasonable modification of the surface can help to achieve the full release of DOX with pH‐responsive function of the carrier.

### 4.5 Metal Oxide Nanoparticles

Iron oxide nanoparticles (IONPs) offer several advantages for applications in therapy and imaging, including their ability to be visualized *via* MRI, their potential for targeted delivery using magnetic fields and their ability to be functionalized. Ali et al. used hematite nanotubes (HNTs) coated with tyramine to reduce systemic toxicity and load the antibiotic meropenem (MP) (Ali et al. 2022). The anti‐bacterial activity of MP@Tyramine‐HNT and MP was compared through the agar disc diffusion method. Results indicated that the anti‐bacterial properties of antibiotics were enhanced after conjugation. The MP@Tyramine‐HNT exhibited a MIC and minimum bactericidal concentration (MBC) of *Klebsiella pneumoniae* better than MP itself, being 8 times and 16 times lower, respectively. In addition, Ebadi et al. synthesized iron oxide (Fe_3_O_4_) nanoparticles using the co‐precipitation method and coated NPs with polyvinyl alcohol, Zn/Al‐layered double hydroxide and the drug sorafenib for cancer treatment (*i.e*., against HepG2 liver cells) (Ebadi et al. 2021). The results using cell viability assays clearly showed that the nanoparticles were more potent than sorafenib alone against HepG2 liver cancer cells, while displaying no cytotoxicity against healthy 3T3 fibroblasts. Iron oxide nanoparticles can be used as catalysts in Fenton reactions to generate cancer destroying reactive oxygen species (ROS), particularly hydroxyl radicals (Ranji‐Burachaloo et al. 2018). The ROS are produced from the electron transfer between iron and hydrogen peroxide (H_2_O_2_), found at high levels within cancer cells. For example, after the nanoparticles are internalized, part of Fe_3_O_4_ decomposes into Fe^2+^/Fe^3+^ in the acidic lysosome and then Fe^2+^/Fe^3+^ diffuses into the cytoplasm, and subsequently, Fe^2+^ reacts with the overproduced H_2_O_2_ to produce O_2_ and ^•^OH. Due to the magnetic properties of iron oxide nanoparticles, they are well studied in biomedical applications involving MRI and magnetic hyperthermia. However, they have been used in other types of therapies such as photothermal, photodynamic, and sonodynamic therapies due to their capability of loading photothermal molecules, photosensitizers and sonosensitizers (Amirshaghaghi et al. 2019; H. Yao and Zhou 2023; P. Zhang et al. 2018). In particular, for photodynamic therapy, the light‐excited photosensitizers can transfer energy directly to O_2_ produced by the Fenton reaction to continuously produce singlet oxygen ROS. In most cases, significantly better inhibitory effects are seen on cancer cells and correspondingly higher levels of intracellular ROS generation, compared with free sensitizers.

Copper oxide nanoparticles (CuO NPs) have been commonly used as anti‐microbial, anticancer and antioxidant agents. Nagore et al. synthesized CuO NPs using *Polyalthia longifolia* leaf extract, exhibiting significant anti‐bacterial effects against various bacterial strains such as *Escherichia coli*, *Streptococcus pyogenes*, *Pseudomonas aeruginosa* and *Staphylococcus aureus* and good anti‐fungal performance against *Aspergillus niger*, *Epidermophyton floccosum*, *Aspergillus clavatus*, and *Candida albicans* (Nagore et al. 2021). The MIC values were from 12.5 to 125 μg/mL for the bacterial pathogens and 100 to 1000 μg/mL for the fungal pathogens. The nanoparticles were more potent as antibacterial agents against *E. coli* and *S. aureus* and more potent as antifungal agents against *C. albicans* and *E. floccosum*. The CuO NPs were more potent than common antibiotics (*e.g*., ampicillin) when used for *E. coli* and *S. aureus*. In a study conducted by Giannousi et al. CuO NPs showed anti‐cancer activity against HeLa cervical carcinoma cells (Giannousi et al. 2016). The IC_50_ values determined were in the 11.91–25.78 μg/mL range, elucidating significant reduction in viability of tumor cells. Membrane damage of cancer cells were a result of ROS production and anti‐inflammatory activity. At a concentration of 100 μg/mL, the antioxidant activities of copper oxide NPs produced using green synthesis methods using spinach leaves extract ((CuONPs)_sp_) and curcumin ((CuONPs)_cur_) were found to be greater (*i.e*.,~92% and 86%, respectively) than that of nanoparticles (*i.e*., 84%) synthesized using a conventional chemical method ((CuONPs)_chem_) (Al‐Jawhari et al. 2022). The IC_50_ value of 21 ± 6 μg/mL for (CuONPs)_sp_ revealed its promising anti‐proliferative effect over (CuONPs)_cur_ and (CuONPs)_chem_, which showed IC_50_ values of 26 ± 3 μg/mL and 60 ± 1 μg/mL, respectively. CuO NPs have also been used as anti‐viral and antidiabetic agents showing a 70% inactivation of viruses after 4 h exposure and 33.66% reduction in blood glucose levels (Cui et al. 2021; Faisal et al. 2022).

Magnesium oxide nanoparticles (MgO NPs) possess several important physiochemical properties like high ionic character, large surface area, unusual crystal morphology and oxygen vacancies, which enable them to easily interact with several biological systems. MgO NPs have been found to exhibit a significant antibacterial effect against both Gram‐positive and Gram‐negative bacterial strains. Pugazhendhi et al. showed that flower‐shaped MgO NPs had strong bactericidal activity against both Gram‐positive (*i.e*., *Streptococcus pneumonia*, MRSA 11, MRSA 56) and Gram‐negative (*i.e*., *Escherichia coli*, *Pseudomonas aeruginosa, Aeromonas baumannii*) bacteria in a dose‐dependent manner (Pugazhendhi et al. 2019). MgO NPs exhibited the highest inhibitory activity against *P. aeruginosa* and MRSA 56 with zones of inhibition 8 and 9 mm, respectively at a concentration of 30 μg/mL. MIC for MRSA 56 and *P. aeruoginosa* was 256 μg/mL while MBC values for MRSA 56 and *P. aeruoginosa* were 256 and 1024 μg/mL of MgO NPs, respectively. At a concentration range of 10–30 μg/mL, the MgO NPs had potent antifungal activity when compared to the positive control (*i.e*., antifungal medication fluconozole). Among the three strains, MgO NPs inhibited the growth of *Fusarium solani* and *Aspergillus niger* more effectively when compared to *Aspergillus fumigates*. MgO NPs showed potent cytotoxic effects against A549 lung cancer cells in a dose dependent manner with an IC_50_ value of 37.5 ± 0.34 μg/mL, in comparison with the positive control cisplatin (*i.e*., IC_50_ value of 25.4 ± 0.024 μg/mL). In another study by Narendhran et al. biosynthesized MgO NPs had higher radical scavenging activity (*i.e*., for 2,2‐diphenyl‐1‐picrylhydrazyl, DPPH), compared to chemically synthesized MgO NPs and control ascorbic acid (Narendhran et al. 2019). The DPPH scavenging activity of biosynthesized MgO NPs was significantly higher with IC_50_ value of 72.24 μg/mL when compared with control ascorbic acid (*i.e*., IC_50_ value of 33.46 μg/mL) and chemically synthesized MgO NPs (*i.e*., IC_50_ value of 66.74 μg/mL). In general, the extracts from plants used to synthesize nanoparticles are rich in several antioxidant components such as chlorogenic acid, caffeic acid, rosmarinic acid, total polyphenols and total flavonoids. These components get incorporated onto nanoparticles from biosynthesis, giving nanoparticles excellent antioxidant and anticancer activities.

### 4.6 Hybrid Nanoparticles

Hybrid nanostructures are constructed from at least two different types of materials, combining the advantages of the materials while mitigating their individual limitations. They provide superior properties not possessed by single component nanostructures, commonly enabling combination therapy and imaging. Hybrid nanoparticles are increasingly recognized for their ability to provide synergistic rather than merely additive therapeutic and diagnostic effects. For example, co‐delivery of two therapeutic agents (*e.g*., drugs, genetic material) can lead to synergistic therapeutic effects and much enhanced treatment efficacy, compared to their single counterparts (Meng et al. 2010; Poon et al. 2015). The concept can be applied with different treatment modalities such as photodynamic therapy, photothermal therapy and chemotherapy (W. Li et al. 2014; X. Yao et al. 2015).

Hu et al. fabricated hybrid magnetic core‐shell gold nanoparticles for drug delivery and controlled release (Y. Hu et al. 2018). The gold nanostars (AuNSs) were successfully embedded intact between an inner silica (SiO_2_) layer and outside mesoporous silica layer to create magnetic core/shell hybrid nanoparticles. The drug paclitaxel (PTX) was then successfully loaded into the mesoporous Fe_3_O_4_@nSiO_2_@AuNSs@mSiO_2_ nanoparticles. The nanocomposites exhibited characteristics of high magnetization, mesoporous nanostructure, photothermal properties and low *in vitro* toxicity. Although coating with silica decreased the saturated susceptibility and photothermal heating effect of the nanoparticles, the hybrid nanomaterials still showed strong magnetization and generated sufficient heat. Due to the incorporation of a thermo‐sensitive medium (*i.e*., tetradecanol, melting point of 39°C) and the AuNSs in the nanocomposites, the amount of drug release could be controlled depending on the irradiation time. Only 2% and 6% of PTX were released from Fe_3_O_4_@nSiO_2_@AuNSs@mSiO_2_ nanoparticles in 100 min at 25°C and 37°C, respectively. At 42°C, 32% release of PTX was detected and more than 37% of the loaded PTX was released under continuous near‐infrared (NIR) laser irradiation for 100 min. Less than 4% was released without NIR irradiation. Due to the photothermal effect from nanoparticles under laser irradiation there was noticeable reduction of cell viability in HeLa cancer cells when treated with the nanocomposites and laser irradiation. After incubation at a concentration of 200 μg/mL for PTX‐loaded Fe_3_O_4_@nSiO_2_@AuNSs@mSiO_2_ nanoparticles and NIR irradiation for 10 min (808 nm, 1 W/cm^2^) the cell viability value was ~25%. Only ~60% cell viability was achieved when cells were treated with 200 μg/mL of Fe_3_O_4_@nSiO_2_@AuNSs@mSiO_2_ nanoparticles (*i.e*., without PTX) and laser irradiation. The viability values of cancer cells were much lower compared to when HeLa cells were treated with PTX‐loaded Fe_3_O_4_@nSiO_2_@AuNSs@mSiO_2_ only (*i.e*.,~85%) at the same experimental conditions.

The use of double hydroxides (LDHs) for biomedical applications has gained attention because of their unique structure and features. LDHs are bi‐dimensional nano‐scaled compositions in which water molecules and anions are dissociated from the positively charged layer of metal hydroxides. They show advantageous physical and chemical features, including thermal stability, catalytic capability, high surface area, anion exchangeability and flexibility and tenability in their interlayer spaces. Ray et al. developed methotrexate (MTX)‐encapsulated polymer (PLGA)‐coated layered double hydroxide nanoparticles for treatment of osteosarcoma (Ray et al. 2017). The nanoparticles significantly inhibited cancer growth in human osteosarcoma bearing mice. Following the administration of bare MTX, the tumor volume was reduced to 1027.1 mm^3^, representing a 33.65% reduction compared to the control group (*i.e*., normal saline). Compared to this, PLGA‐MTX showed a better antitumor effect than bare MTX with a mean tumor volume of 580.2 mm^3^, representing 62.53% reduction compared to the control and 43.52% reduction in tumor volume compared to bare MTX. Importantly, PLGA‐LDH‐MTX showed even better results in reduction of the mean tumor volume to 290.86 mm^3^, representing a substantial reduction (*i.e*., 81.26%) compared to the control and around 50% reduction compared to PLGA‐MTX. Compared to bare MTX, the transport effectiveness of MTX by utilizing the hybrid system was remarkably enhanced and thus showed higher accumulation in the targeted cancer tissue. After 4 h from administration, the MTX concentration in tumor tissues from PLGA‐LDH‐MTX was ~162 μg/g, higher than that from PLGA‐MTX (*i.e*., ~ 100 μg/g) and MTX (*i.e*., ~ 87 μg/g) when the same dose of MTX drug was used for formulations (*i.e*., 30 mg/kg MTX).

## 5 Diseases or Conditions in Which BNDSs Are Commonly Applied for Diagnosis and Therapy

BNDSs can be used to treat a variety of infectious and chronic diseases due to their anti‐bacterial, anti‐fungal, antiviral, antidiabetic, anticancer and anti‐inflammatory effects. Infectious diseases associated with bacteria, yeast, fungi and viruses can be treated using inorganic nanoparticles that are more effective than commonly used antibiotic, anti‐fungal and antiviral medication. Chronic diseases such as cancer, Alzheimer's disease, Parkinson's disease and diabetes can also be treated using BNDSs. In particular, nanoparticles produced using natural compounds such as from plants are more potent for therapy than plant extracts alone.

### 5.1 Infectious Diseases

Infectious diseases are becoming a global concern and bioinorganic nanoparticles can be employed as effective antimicrobial delivery agents. The challenge of the development of resistance of pathogenic microorganisms to antimicrobials has become an impediment towards the successful diagnosis and treatment of pathogenic diseases. Nano‐sized metal particles have proved to be a promising alternative to antimicrobials owing to their ideal properties, capable of preventing problems associated with the development of multidrug resistance in pathogenic microorganisms (Naseem et al. 2024). Some of these nanoparticles possess antimicrobial effects and hence improve therapeutic outcomes.

Research on AgNPs has gained attention and is thought to have multifunctional bio‐applications including antibacterial, antifungal, antiviral, anticancer and anti‐inflammatory effects (X.‐F. Zhang et al. 2016). AgNPs exhibit multifaceted mechanisms of action toward the destruction of bacteria and the most common modes of action include modulation of microbial signal transduction pathways, generation of free radicals and ROS. Furthermore, the ability of AgNPs to adhere and penetrate inside microbial cells enhances cytotoxicity. Studies have demonstrated the effective use of AgNPs in delivering antibiotics in a controlled manner, facilitating the reduction of side effects at lower dosages and circumventing the development of drug resistance in the process (Dakal et al. 2016).

Metallic nanoparticles can be employed as effective anti‐viral agents either in their original or modified form. The formulation methods used are important as they affect the morphology, size of the particles, particle charge and surface chemistry exhibited by the nanoparticles, influencing their antiviral activities. Several dosages can be utilized, using aqueous dispersions to encapsulate composite forms. AuNPs possess ideal properties such as small size, high surface area‐to‐volume ratio, biocompatibility and potential for drug delivery, making them promising candidates for antiviral drug development. They have the potential to deliver significant amounts of drug across biological barriers for the management of viral infections such as COVID‐19 (T. Y. Hu et al. 2020; S. Paul et al. 2023; Raji et al. 2020).

Caniglia et al. fabricated AgNPs conjugated with catecholamine‐based polymer polydopamine (PDA) to investigate their anti‐microbial effects on *Escherichia coli* (Caniglia et al. 2024). PDA was incorporated as a coating of AgNPs to enhance their stability. Results demonstrated loss of stiffness and enhanced hydrophilicity of the *E. coli* envelope when bacteria were in close proximity (2–5 μm) to the AgNPs. The antimicrobial activity was attributed to the release of silver ions from NPs.

Bernardo et al. investigated the antimicrobial effects of lyophilized plant extracts of *Syzygium cumini* (HEScSeed and HEScFlower) and AgNPs (AgNPs‐HEScSeed and AgNPs‐HEScFlower) of *S. cumini* seed and flower (de Carvalho Bernardo et al. 2021). Results revealed that AgNPs‐HEScSeed and AgNPs‐HEScFlower exhibited different properties related to their size, stability, electronegativity, shape and different organic compounds present. These differences led to different fungistatic and bacteriostatic effects. The study confirmed activity against *Candida albicans* (*C. albicans*), *Staphylococcus epidermidis* (*S. epidermidis*) and *Staphylococcus aureus* (*S. aureus*) among others. Significantly lower concentrations were effective compared to plant extracts alone and the MICs were species dependent. Smaller MIC values were presented for AgNPs compared to the plant extracts. The MIC values for HEScSeed and AgNPs‐HEScSeed extracts were 1296.8, 648.4 and 648.4 µg/mL and 2000, 31.2, 125 µg/mL against *C. albicans*, *S. epidermidis* and *S. aureus*, respectively. Results followed the same trend for the HEScFlower and AgNPs‐HEScFlower, where the MIC values were 2593.7 µg/mL against *C. albicans*, *S. epidermidis* and *S. aureus* when treated with HEScFlower and 250 µg/mL against *C. albicans*, *S. epidermidis* and *S. aureus*, when treated with AgNPs‐HEScFlower.

A study by Asghar et al. analyzed iron, copper and silver nanoparticles, synthesized using *S. cumini* leaf extract, for their anti‐microbial activities against methicillin‐resistant *S. aureus* (MRSA) and vancomycin‐resistant *S. aureus* (VRSA) infectious strains (Asghar et al. 2020). The study showed improved anti‐microbial activity by using AgNPs. Inhibition zones in the 11–20 mm range and MIC values in the 8–128 µg/mL range for were determined for NPs. Furthermore, aflatoxins produced by *Aspergillus flavus* and *Aspergillus parasiticus* was reduced more than 40% from the use of NPs, showing that the NPs can also be used for their antifungal activity. Hence data proved that metal NPs synthesized from *S. cumini* have potential medical and pharmaceutical applications.

Baveloni et al. investigated the potential of AgNPs in wound healing (Baveloni et al. 2025). Results showed bactericidal activity against *S. aureus* and *P. aeruginosa* at a concentration of 6.74 µg/mL, with a much higher concentration (58.5 µg/mL) required for bactericidal activity against *E. coli*. Animals treated with AgNPs exhibited 90% re‐epithelialization along with effective microbiological control. A significant reduction in bacterial counts after 72 h of 99.9% was demonstrated at a MBC of 13.5 µg/mL for *S. aureus* and *P. aeruginosa*, indicating enhanced microbiological control. Depending on whether infectious pathogens are Gram‐positive or Gram‐negative bacteria, the results varied in bacterial susceptibility, potentially due to differences in metabolic pathways and cell wall structure. Research conducted on AgNPs‐coated nylon sutures revealed bacteriostatic effects with more than 90% reduction in bacterial growth. In addition, the haematoxylin and eosin staining of buccal mucosa demonstrated that AgNPs‐coated sutures attenuated inflammatory cells at wound sites (Syukri et al. 2024).

Mumtaz et al. synthesized silk fibroin‐chitosan blend zinc oxide (ZnO) NPs for assessing their anti‐microbial effect on Gram‐negative and Gram‐positive bacteria (Mumtaz et al. 2024). ZnO NPs possess bactericidal properties and have high capacity for adsorption, which makes them favorable nanoparticles (Malik et al. 2024). They elucidated the broad‐spectrum antibacterial properties against harmful microbes. The release of zinc ions after internalization of NPs leads to oxidative stress, due to the production of ROS. Hydrogen peroxide is a strong oxidizing agent giving rise to cell membrane destruction and disturbances in cell metabolism, leading to bacterial death. The results of the study demonstrated the highest zone of inhibition against all strains of bacteria at the highest concentration of 8 mg/mL of silk fibroin‐chitosan blend ZnO NPs. ZnO NPs showed significant antibacterial effects on Gram‐negative bacteria such as *Klebsiella pneumoniae*, *Serratia rubidaea*, *E. coli* and *Proteus mirabilis* (*i.e*., zones of inhibition of 11.3 ± 0.9 mm, 16.7 ± 0.9 mm, 12.7 ± 0.9 mm, 11.7 ± 1.2 mm, respectively) and Gram‐positive bacteria such as *S. aureus, Bacillus thuringiensis* and *Clostridium difficile* (*i.e*., zones of inhibition of 17.3 ± 1.2 mm, 12.3 ± 0.3 mm, 11.7 ± 1.2 mm, respectively).

In another study by Al‐Radadi et al. ZnO‐NPs were engineered to enhance the efficacy of conventional antibiotics such as amoxicillin‐clavulanic acid, ciprofloxacin, imipenem and vancomycin against *S. aureus* and *E. coli* (Al‐Radadi et al. 2022). Compared to conventional antibiotics there was an increase in anti‐microbial activity from the use of ZnO‐NPs coated with antibiotics. Results showed enhanced activity against *S. aureus* and *E. coli* up to 10.5%, 11.2%, 24.1%, 35.3% and 15.8%, 23.9%, 31.8%, 41.4%, for vancomycin, amoxicillin‐clavulanic acid, imipenem, and ciprofloxacin, respectively.

Ontong et al. synthesized AgNPs using *Rhodomyrtus tomentosa* leaf extract and silk sericin to functionalize carbopol 940 gel for topical applications (Ontong et al. 2020). AgNPs demonstrated broad‐spectrum anti‐microbial activity against Gram‐positive, Gram‐negative, and fungi with MIC ranging between 0.26 and 2.10 µg/mL. The gel showed spreadability and extrudability of 9.3 ± 0.85 s and 19.85 ± 0.03%, respectively.

### 5.2 Noncommunicable Diseases

The leaky vasculature and poor lymphatic drainage associated with cancer enable nanosized metal particles such as AuNPs and AgNPs to passively accumulate at the tumor site. These NPs are often used in oncology to deliver chemotherapeutic agents to cancer cells, enhancing cytotoxicity and reducing systemic side effects. The ability to tune the size of these particles, and their versatile surface characteristics, allow for conjugation of metal particles with significant amounts of imaging agents for cancer (Fernandes 2023d, 2024b; Prajapati et al. 2024). AuNPs play a role in photothermal therapy in which laser irradiation causes localized heating, leading to the destruction of cancer cells (Chen et al. 2016).

Superparamagnetic iron oxide nanoparticles (SPIONs) and other iron NPs have been investigated for use in MRI and demonstrate a higher contrast and biocompatibility, compared to many other nanoparticles (Fernandes 2023a). Nanoporous silica nanoparticles have been reported to display enhanced signal strength as well as good localization with cancer cells as observed under MRI. Mesoporous silica nanoparticles (MSNs) have recently been developed as flexible imaging platforms owing to their useful physicochemical properties, tuneable size, and surface chemistry. They have also commonly been used as carriers for drug delivery (Yuan et al. 2020). The addition of ligands on the surface of NPs are used to recognize cancer cell markers, enhancing treatment outcomes for patients (Jin et al. 2024). Tay et al. demonstrated greater effectiveness of superferromagnetic iron oxide nanoparticles (SFMIOs) as a noninvasive imaging agent for cancer, pulmonary embolism, inflammation and gastrointestinal bleeds, compared to conventional magnetic particle imaging (MPI) SPIONs (Tay et al. 2021). The novel SFMIOs showed improvement in spatial resolution and signal‐to‐noise ratio in MPI experiments. The use of SFMIO NPs led to 10‐fold improvement in spatial resolution and 40‐fold stronger signals, compared to conventional SPIONs.

Liu et al. developed a silica‐based nanoplatform with potential for theranostic applications in lung cancer management (Liu et al. 2020). The multifunctional nanosystem encapsulated a chemotherapeutic agent and magnetic cores and was coated with an antibody against lung cancer stem cells (CSC). The administered NPs are activated by an externally applied magnetic field (AMF). *In vitro* studies showed up to 98% lung CSC death with 30 min of application of the AMF due to the combined effects of hyperthermia and chemotherapeutic drug treatment. *In vivo* studies demonstrated significant suppression of tumor growth and metastasis in lung CSC xenograft‐bearing mice with minimal side effects.

Wang and Sun constructed a novel theranostic drug conveyance device aimed at effectively treating breast cancer (L. Wang and Sun 2020). Gadolinium(III) oxide nanoparticles (Gd‐NPs) and ultra‐iron nanoparticles (uFe‐NPs) were used to prepare superparamagnetic Gd‐NPs with multiscale sizes. Gd‐NPs acted as an alternate magnetic field‐responsive heat mediator whereas uFe‐NPs acted as an MRI contrast mediator. Mesoporous silica sphere with radially directed mesochannels was further developed *in situ* on the surfaces of Gd‐NPs, with both uFe‐NPs and biotin (Bt) easily incorporated. *In vitro* studies revealed a higher cytotoxicity towards MCF‐7 cancer cells. For the Gd‐MSN@uFe‐Bt‐NPs group exposed to AMF for 15 min, a further effective anticancer consequence was observed with a lower cell viability of 45% after 48 h of incubation. Results further demonstrated that Gd‐MSN@uFe‐Bt‐NPs had a high potential for cancer tissue targeting, for improving *in vivo* chemotherapy.

Medications used for the treatment of neurodegenerative diseases such as Parkinson's disease and Alzheimer's disease (AD) need to be able to cross the blood‐brain barrier (BBB) to effectively target the central nervous system. The potential of BNDSs to penetrate the BBB is being investigated. Magnetic nanoparticles have been used to improve drug delivery to the central nervous system (CNS). Patients with AD have increased expression of pro‐inflammatory cytokines in the nervous system leading to impaired neuron function. AuNPs have emerged as agents with promising physicochemical properties and anti‐inflammatory effects, utilized for reducing neuro‐inflammation by inducing macrophage polarization towards the M2 phenotype (Aili et al. 2023). Studies have been conducted to investigate the potential use of AgNPs for prophylaxis of infections postsurgery and managing associated inflammation (Ontong et al. 2020; Syukri et al. 2024). By using an external magnetic field, to drive magnetic NPs toward specific regions of the brain, localized treatment of diseases such as Alzheimer's and Parkinson's diseases can be made possible. Current research indicates the potential applications of BNDSs for neural and cardiovascular conditions. For example, silica nanoparticles have shown promise in delivering therapeutic agents for neuro‐inflammation whereas other NPs deliver drugs that target myocardial infarctions and atherosclerosis sites (Gong et al. 2023; C. Wang et al. 2023).

CD163 is a membrane receptor usually overexpressed at inflammatory sites. Tarin et al. developed a sensitive probe for CD163 detection in atherosclerosis using MRI. A targeted probe based on gold‐coated iron oxide nanoparticles tagged with an anti‐CD163 antibody for the specific detection of the membrane receptor was prepared. Results indicated selective detection of CD163(+) macrophages both in humans and murine cells (Tarin et al. 2015). MRI imaging postinjection of the targeted probes in 16‐week‐old mice developing atherosclerotic lesions showed an increase in accumulation at the region of interest over time and the signal intensity decreased significantly 48 h after injection. Hence the probe can be used for investigation of the state of atheromatous lesions.

A study by Al‐Radadi et al. utilized *Zingiber officinale* extract for the reduction and capping of ZnO‐NPs, demonstrating potential for inhibition of Alzheimer's disease (AD), diabetes and reduction of inflammation (Al‐Radadi et al. 2022). ZnO‐NPs showed significant inhibition of α‐amylase up to 51.12 ± 0.71% at 400 µg/mL, showing anti‐diabetic potential. The study indicated the potential of ZnO‐NPs for treating Alzheimer's disease at a concentration of 400 µg/mL, by inhibiting acetylcholinesterase (AChE) and butyrylcholinesterase (BChE) up to 62.91 ± 1.62% and 60.18 ± 0.21%, respectively. The nanoparticles inhibited cyclooxygenase (*i.e*., COX) at 400 µg/mL concentration by 53.11 ± 1.12% for COX‐1 and 51.14 ± 1.16% for COX‐2 showing their anti‐inflammatory activity.

In another study, researchers synthesized lipid‐coated mesoporous silica nanoparticles containing berberine (MSNs‐BBR‐L) for effective treatment of Alzheimer's disease (Singh et al. 2021). MSNs‐BBR‐L displayed enhanced AChE inhibitory activity. The study confirmed significant amyloid fibrillation inhibition and decreased malondialdehyde levels. These results highlight the use of MSNs‐BBR‐L as promising drug delivery vehicles to the brain as the NPs can cross the BBB. BACE‐1 is an enzyme responsible for producing amyloid‐beta (Aβ) peptides that are increased in levels in patients with AD. MSNs‐BBR‐L treated AD animals showed a significant decrease in BACE‐1 levels, compared to scopolamine‐intoxicated mice.

Alomari et al. conducted a study to provide insight into the effects of AuNPs on diabetic neuropathy which is a major contributing factor towards end‐stage renal failure in patients with diabetes (Alomari et al. 2020). Adult male rats were used for the study where three groups were investigated. The groups used were the nondiabetic control group, the diabetic group and the diabetic group treated intraperitoneally with 50‐nm AuNPs (*i.e*., at 2.5 mg/kg/day for 7 days). A single dose of streptozotocin (55 mg/kg) was used to induce diabetes in the experimental rats. Results showed that treatment with AuNPs in rats prevented diabetes‐associated rise in glucose levels with reduced 24‐h urinary albumin excretion rate, glomerular basement membrane thickness and renal oxidative stress markers. Furthermore, the results revealed the downregulation effect of AuNPs in renal mRNA or protein expression of transforming growth factor β_1_ (TGF‐β_1_), fibronectin, tumor necrosis factor‐α (TNF‐α), and vascular endothelial growth factor‐A (VEGF‐A). Hence there is evidence from the study that 50‐nm AuNPs can reduce kidney damage in experimental models of diabetic neuropathy through improving renal function.

Dul et al. attached an autoantigen to AuNPs for the treatment of Type 1 diabetes (PIc_19‐A3_ peptide), creating negatively charged AuNPs‐peptide complexes (Dul et al. 2019). A microneedle delivery system (MicronJet600) was employed to facilitate minimally invasive intradermal delivery of the NPs. The platform was constructed to target skin‐resident antigen presenting cells which are known to be apposite target cells for immunotherapy. *In vitro* studies showed that the uptake of AuNPs‐peptide complexes by dendritic cells reduced the capacity of these cells to activate naïve T‐cells. This was an indicator of biological functionality, which encourages further development of the AuNPs‐peptide formulation. Clinical studies are underway to investigate the potential of this nanoplatform in targeted immunotherapy.

## 6 Clinical Trials Using BNDSs

Clinical studies have incorporated a wide range of particle‐based nanosystems for use as nanomedicine. Each type of inorganic nanoparticle can be used in unique applications for treatment and diagnosis, with some having more challenges than others, preventing their approval by agencies such as FDA and EMA.

Several iron‐based nanoparticles have been explored in clinical trials as magnetic hyperthermia agents against cancer, for treating anemia and for MRI (*e.g*., for atherosclerosis, heart disease, inflammation, diabetes) in diagnosis/monitoring. Magnablate is a suspension containing iron oxide nanoparticles, developed for magnetothermal therapy of prostate cancer and that has been used in a phase 0 clinical trial (NCT02033447). NanoTherm (aminosilane‐coated SPIONs) has been approved by EMA for glioblastoma treatment *via* local hyperthermia (Thiesen and Jordan 2008). After the local injection into tumors, the nanoparticles are selectively heated by an alternating magnetic field applicator to achieve local temperature elevation of the tumor environment to 40°C−45°C. This results in programmed and nonprogrammed cell death. In clinical trials, therapy of glioblastoma tumors with NanoTherm exhibited an overall survival increase of up to 12 months (Maier‐Hauff et al. 2011). FDA approval for clinical testing using these nanoparticles to treat prostate cancer has been received. Interstitial heating using these nanoparticles was found to be feasible in patients with previously irradiated and locally recurrent prostate cancer (Johannsen, Gneveckow, Thiesen, et al. 2007). The major advantage of the approach utilizing magnetic nanoparticles for hyperthermia is that the magnetic fields can be localized at tumor sites to avoid damage to healthy tissues. In addition, therapeutic agents can be attached to nanoparticles for targeted combination treatment. Having said this, the majority of cancers are metastatic, spreading to various parts of the body and cannot be individually targeted. Sufficiently high concentrations of nanomaterials are required at tumor areas to achieve significant thermal ablation or hyperthermia. It is necessary to further develop iron‐based therapies with optimized heat‐generation properties and bio‐distribution profiles for enhancing therapeutic efficacy. Other iron‐based nanoparticles such as EMA approved dextran‐coated iron oxide nanoparticles (Sienna^+^) have been used in a phase IV clinical trial for detection of cancerous sentinel lymph nodes in breast cancer patients (NCT02612870). Gastromark/Lumirem/Ferumoxsil/AMI‐121 and Abdoscan/Ferristene/OMP are formulations containing siloxane‐coated iron oxide nanoparticles and polystyrene‐coated iron oxide nanoparticles, respectively, for MRI of the gastrointestinal tract. A variety of iron‐based formulations have been extensively studied for treating iron deficiency, kidney disease and heart conditions such as CosmoFer/INFeD/Ferrisat, DexFerrum/DexIron, Ferrlecit, Venofer, Injectafer/Ferinject, and Monofer (Auerbach and Macdougall 2014; Bansal et al. 2010; Baribeault 2011; Geisser and Burckhardt 2011; Hood et al. 2000; Kalra and Bhandari 2016; L. J. Scott 2018). Table 2 presents r_1_ and r_2_ relaxivities of some magnetic NPs used in clinical trials, for positive and negative contrast, respectively.

**Table 2:** The r_1_ and r_2_ relaxivities of commonly used SPIONs as a measure of efficiency of MRI enhancement (Knobloch et al. 2018; Y.‐X. J. Wang 2011).

Generic name	Brand name	r_1 (mM_^‐1^_s_^‐1^)	r_2_ (_mM_^‐1^_s_^‐1^)	Primary clinical application
Ferumoxtran‐10	Combidex	10	60	Lymph node metastasis imaging
Ferumoxytol	Feraheme	19	60–65	Iron deficiency anemia; off‐label for vascular/cardiac imaging
Ferumoxides	Feridex IV	23.9	98.3	Liver and spleen imaging
Ferucarbotran	Resovist	25.4	151	Liver imaging

Noble metal‐based nanoparticles have gained attention in clinical studies due to their unique photothermal properties, ability to precisely tune the size and shape and adjust surface properties by means of conjugation with various antibodies, nucleic acids, polymers and small‐molecule therapeutics. Paciotti et al. constructed a gold nanoplatform modified with recombinant human tumor necrosis factor (rhTNF), for solid tumor therapy (Paciotti et al. 2004). The phase I clinical trial of CYT‐6091 was carried out on patients suffering from advanced or refractory malignant solid tumors (NCT00356980) (Libutti et al. 2010). Patients were intravenously injected with escalating dosages of CYT‐6091 with only 62% of patients receiving treatment observed with transient moderate hypotension. Even at the highest dose rhTNF's dose limiting toxic effect of hypotension was need seen. Findings suggested that CYT‐6091 can effectively target tumor sites. In a phase II clinical trial, patients with nonsmall lung cancer were given CYT‐6091 first, followed by standard chemotherapy, to assess the safety and efficacy of combining CYT‐6091 with approved chemotherapies. AuroLase involves silica‐gold nanoshells, coated with polyethylene glycol (PEG) that has been developed for photothermal ablation of solid tumors, with stimulation by NIR irradiation (Rastinehad et al. 2019; Stern et al. 2016). In a pilot study AuroLase was combined with MRI‐ultrasound imaging for visualizing the photothermally ablated cancerous tumors within the prostate. The study underlined the feasibility and safety evaluation of AuroLase within patients subjected to ablation of low‐ or intermediate‐grade focal prostate cancer. After infusion of AuroLase particles and high‐precision laser ablation, patients underwent multiparametric MRI of prostate tumors, followed by post‐procedure multiparametric high‐resolution MR imaging/ultrasound targeted fusion biopsies with standard 12‐core systematic biopsy. Overall, AuroLase‐directed focal laser ablation proved considerably effective for 94% of patients without significant disparity in prostate symptoms or health of men after treatment. AuroLase provides localized tumor therapy, avoiding systemic side effects correlated with traditional cancer therapy. However, this type of therapy using NIR has limited tissue penetration depth, making it difficult to effectively treat deeper tumor tissues within the body. Silver nanoparticles have also been used in clinical trials such as SilvaSorb (*i.e*., a kind of antibacterial silver nanoparticle gel). A phase III trial tests the efficacy of SilvaSorb against the standard antibacterial hand gel (*i.e*., Purell), by comparing the survival rate of the surrogate biomarker *S. Marcescens* on the patients' hands after a single utilization of either gel (NCT00659204). Even though there has been substantial research on the biomedical applications of silver nanoparticles, their biosafety still requires to be further investigated, for example their influence on the immune response on the lungs and possible absorption in the bloodstream. The bio‐distribution, pharmacokinetics and biological effects from prolonged exposure of other noble metal‐based nanoparticles need to be further studied as the nanoparticles may cause irreversible damage (*e.g*., pigmentation of the skin or eyes).

Other inorganic metal‐based nanoparticles used in clinical trials include hydroxyapatite and calcium phosphate nanoparticles, titanium dioxide nanoparticles, zinc oxide nanoparticles and gadolinium‐based systems. A clinical study was conducted to compare outcomes of a nanocrystalline hydroxyapatite (Ostim) and an enamel matrix derivative (Emdogain) in regenerative periodontal treatment (NCT00757159) (Al Machot et al. 2014). Both groups showed significant bone fill, reduction of probing pocket depth, increase in recession and gain of attachment. No significant differences between groups were found at any time point. However, the enamel matrix derivative could have some advantages compared to the nanocrystalline hydroxyapatite regarding patient comfort and adverse events. There are several calcium phosphate (CaP)‐based products on the market as artificial bone grafts, such as Vitoss, OsSatura, NanOss, and EquivaBone. There are two CaP based materials in clinical trials, utilized for bone resorption (Nano Bone, NCT03980847) and maxillary sinus lift (Nano Streams, NCT03177876). Although the composition of CaP‐based bone graft substitutes is roughly the same as that of natural bone, they lack the organic components of natural bone. More attention should be on the use of biomimetic CaP‐based nanosystems. Titanium dioxide nanoparticles have been used for treating *Candida* infection, denture stomatitis and dental anxiety (NCT02950584). For clinical progress in the use of titanium oxide nanoparticles, detailed toxicokinetic studies must be conducted, including the absorption, distribution, metabolism, accumulation and excretion of these nanoparticles that enter the body through different exposure pathways. The systemic responses and biomarkers must be assessed that reflect the toxic effects of nanoparticles. The molecular mechanisms associated with the therapeutic response from treatment with nanoparticles must be elucidated. Clinical studies have taken place involving zinc oxide nanoparticles for treating foot dermatoses and dental caries (NCT04000386, NCT03478150). Zinc oxide nanoparticles share similar key issues with many other metal oxide nanoparticles due to insufficient *in vivo* toxicity studies. Gadolinium (Gd)‐based contrast agents have been used for MRI. There are mainly two families of commercially available Gd‐based contrast agents, one using linear complexing molecules (*e.g*., MAGNEVIST, OMNISCAN, OPTIMARK, MultiHance) and the other employing macrocycles structures (*e.g*., PROHANCE, GADAVIST, DOTAREM). However, these Gd‐based molecular complexes have fundamental limitations including residual toxicity and unsatisfactory efficiency. This has led to the development of Gd‐based inorganic nanoparticles (*e.g*., AGuIX, polysiloxane Gd‐chelates based nanoparticles) with improved pharmacokinetics, avoiding both significant release of free Gd ions and significant systemic toxicity while improving contrast for imaging (Bianchi et al. 2015; Sancey, Motto‐Ros, et al. 2014). AGuIX nanoparticles have been used in pre‐clinical and clinical studies, demonstrating very high radiosensitizing properties (*i.e*., for radiation therapy) together with excellent MRI positive contrast properties due to the paramagnetic properties of Gd (NCT02820454, NCT03308604) (Kotb et al. 2016; Lux et al. 2019; Sancey, Lux, et al. 2014).

## 7 Challenges With BNDSs

The development of inorganic nanoparticles for therapy and diagnostics has significantly grown yet there still remains significant biological, technological and study‐design‐related challenges. Biological challenges include their fast immune clearance, difficulties navigating biological barriers (*e.g*., blood‐brain, mucosal, epithelial, intracellular, tumor microenvironment, enzyme‐related, pharmacokinetic‐related) and potential toxicity, inflammation responses and oxidative stress, which can lead to cell damage and dysfunction in normal, healthy cells (Waheed et al. 2022). Many previous experimental studies have shown that nanoparticles can harm organs and physiological systems. For example, many inorganic nanoparticles can disrupt the membrane structure, perturb the mitochondrial function and elicit nuclear alterations, resulting in different types of cell deaths (*e.g*., necrosis, apoptosis, autophagy, ferroptosis) (Xuan et al. 2023). Toxicity effects are dependent on the physicochemical properties, dosage, exposure duration, administration route and the amount of toxic ions released upon exposure to nanoparticles (Missaoui et al. 2018). Some factors to consider when designing nanoparticles that govern toxicity are surface area, particle size and shape, solubility and agglomeration. Fabricating nanoparticles with more biocompatible substances and materials can reduce their toxicity. Most nanoparticles including inorganic types cannot avoid the mononuclear phagocytic system, a network of cells involved in immune responses and tissue homeostasis. When traveling in the circulatory system, nanoparticles adsorb proteins, forming a protein corona on its surface. This marks nanoparticles for immediate clearance by phagocytic cells, making them ineffective in treating the targeted disease site. Advanced strategies are required in engineering the surface of nanoparticles to significantly reduce the number of proteins adsorbed for preventing macrophage recruitment.

Technological challenges relate to obstacles in scaling up quantities, optimization of experimental parameters and performance predictions. For example, it might not be feasible to produce large amounts of some nanoparticles given instrumentation and product costs for synthesis. Systemic approaches are required for product development and manufacturing for optimization of different parameters such as zeta potential, particle size, entrapment efficiency, polydispersity index and drug release using statistical experimental design (Birla et al. 2025). The incorporation of such approaches can reduce costs, minimize batch failures and improve product consistency, reducing the time taken for the product to arrive on the market.

Computational or theoretical modeling can be used along with experimental work to enhance results and improve predictions of nanoparticle efficacy and performance before clinical trials. Study‐design challenges like the size of studies, intent and timings for treatments using nanoparticles can influence the results. The genetic and environmental factors and past medical history of the patient are important in whether the nanoparticles are successful in treating the disease (Schork 2015). Hence, the nanoparticles should be designed for usage in personalized medicine with appropriate cell and animal models.

Unified international standards and testing methodologies are required for standardization, accurate characterization of nanoparticles and product development. This will ensure the safe, effective, and responsible application of inorganic nanoparticles as therapeutic and diagnostic agents. The utilization of inorganic nanoparticles in the biomedical field is full of opportunities but collaboration between the scientific community, industry and regulatory authorities is required for humanity to benefit substantially from its capabilities.

## 8 Current Trends to Overcome the Challenges Associated With BNDSs

A variety of methods have been proposed and successfully applied for improving the theranostic efficacy of BNDSs. Two of the most important methods include designing nanoparticles to be responsive to external conditions or specific microenvironment and/or intracellular conditions and surface modification for improving biocompatibility and targeted delivery.

### 8.1 Smart Stimuli‐Responsive Formulations

Understanding the environment in which nanoparticles should be used for delivery to achieve the intended theranostic purposes is crucial in overcoming the challenges associated with their use. Technological advancements in the field of pathophysiology have assisted in understanding the microenvironments of diseased tissues. Metallic nanoparticles can be designed as intelligent formulations, positively affecting disease pathologies and reducing some of the challenges related to their applications in disease management. In terms of drug delivery for diseases such as cancer, BNDSs can respond to external stimuli such as light, magnetic fields and temperature and internal stimuli such as redox reactions, pH, and enzymes specific to the microenvironment or cells targeted. Moreover, the excellent properties of bioinorganic NPs, such as responsiveness to stimuli, can be used to design formulations capable of eliminating cancerous cells. Numerous studies have reported the photothermal therapy potential of AuNPs in cancer treatment wherein light absorbed by AuNPs is converted into heat energy.

### 8.2 Bio‐Functionalization

An understanding of the microenvironments of tumors has led to the identification of specific cell receptors that are unique to specific diseases. Designing nanosystems functionalized with ligands to target these receptors termed ligand‐targeted delivery can overcome some of the challenges faced by bare inorganic nanosystems. For maximizing theranostic capabilities of bioinorganic nanomaterials, cellular uptake is of utmost importance considering that diseases involve molecular alterations in normal cellular function. Compared to organic nanomaterials, inorganic nanomaterials can be easily bio‐functionalized for improved interactions with specific receptors of cells (Unnikrishnan et al. 2023). Alteration of nanoparticle surface characteristics via bio‐functionalization allows for improved interaction for enhancing cellular uptake. In addition to improved cellular uptake, bio‐functionalization improves biocompatibility, important for targeted drug delivery and for reducing the uptake of nanomaterials by the reticuloendothelial system. Bio‐functionalization can be achieved through the addition of functional groups or small ligands that are bonded to the surfaces of nanoparticles that drive entry into the cell. Moreover, the high surface area‐to‐volume ratio of inorganic nanoparticles provides the ability to load significant amounts of agents using bio‐functionalization, maximizing their ability for targeted drug delivery (Fernandes 2022b; Juma et al. 2024).

## 9 Conclusions

Inorganic nanoparticles have shown great promise in the biomedical field due to their unique physicochemical properties such as high surface area‐to‐volume ratio and superior optical, magnetic and electronic properties. This enables them to serve as important platforms for bio‐imaging and disease diagnosis and treatment through functionalization, for overcoming the limitations present in conventional therapy (*e.g*., adverse effects from systemic toxicity). The properties of inorganic nanoparticles can be tuned to meet treatment requirements. For example, for antitumor therapy, the size, shape and surface properties of nanoparticles can be modified to enhance their distribution and residence time within the tumor microenvironment. This can lead to improvements in treatment efficacy. The development and use of advanced technologies that are highly sensitive toward detecting inorganic nanoparticles have proven to be valuable in preventing significant health hazards. These methodologies enable researchers to determine the precise location(s) of nanoparticles and their amount(s) *in vivo* by analyzing high‐resolution images of exposed organs and tissues.

The rapid development of different types of inorganic nanoparticles for diagnostics and therapy has been due to the recent emergence of a few research directions. The number of studies on the safety and efficacy of inorganic nanoparticles in preclinical animal models and clinical trials have increased over the years, important for establishing standardized nanotherapeutic platforms (H. Huang et al. 2020). Preclinical experiments are crucial as the toxicological effects of many types of inorganic nanoparticles are still unclear. Artificial intelligence (AI) and machine learning (ML) are revolutionizing the pharmaceutical industry, as these technologies enable for precision medicine (Noury et al. 2025). Extensive data sets are analyzed to optimize formulations and predict patient responses. AI‐driven models enhance nanoparticle‐based drug carriers, improving their stability, bioavailability and targeting accuracy. ML also facilitates real‐time monitoring and adaptive control of drug release, ensuring better therapeutic outcomes. The integration of AI and ML in drug delivery has the potential of accelerating development and reducing costs. Softer nanoparticles, particularly those that are bio‐inspired and contain biocompatible materials like polymers or lipids, have been found to be able to better evade the immune system because their surface properties are more similar to those of native proteins and cell membranes (Zhang et al. 2022). Biomimetic nanoparticles include inorganic nanoparticles coated with natural membranes (*e.g*., membranes of tumor cells, red blood cells, platelets, immune cells, mesenchymal stem cells, bacterial cells, exosomes). These nanoparticles have extended blood circulation times for treating the target site.

## Funding

The authors have nothing to report.

## Ethics Statement

The authors have nothing to report.

## Conflicts of Interest

The authors declare no conflicts of interest.

## Data Availability

Data sharing not applicable to this article as no data sets were generated or analyzed during the current study.
